# Vegetarianism

**Published:** 1870-04

**Authors:** 


### VEGETARIANISM.

A VEGETARIAN one day asserted that if a man ate beef he would soon have the nature of an ox; would look sheepish if he ate mutton, and become a pig, if he ate pork. "But," said Dr. Walker, "if a man lives wholly on vegetables, he will, on the same principle, be pretty apt to become small potatoes."

Laying jesting aside, the great, broad fact is indisputable, that the great nations of the earth, those who live in the North Temperate Zone, and who make the products of all climes tributary to their tables, which are bountifully spread with the meats, and fish, and fruits, and vegetables of all lands and seas, are the greatest in the aggregate, while individually they have produced the greatest names in all history.

